# Recurrent Chest Pain as the Sole Initial Manifestation of Acute Cholecystitis: A Case Report and Diagnostic Challenge

**DOI:** 10.7759/cureus.106560

**Published:** 2026-04-07

**Authors:** Bassem Al Hariri, Humam Rajha, Osama Alkeilani, Baha H Abuajameia, Ahmad Alharafsheh

**Affiliations:** 1 College of Medicine, Qatar University, Doha, QAT; 2 College of Medicine, Weill Cornell Medicine - Qatar, Doha, QAT; 3 Department of Internal Medicine, Hamad Medical Corporation, Doha, QAT; 4 Department of Pharmacy, Hamad Medical Corporation, Doha, QAT

**Keywords:** acute cholecystitis, acute coronary syndrome, chest pain, diagnostic imaging, referred pain

## Abstract

Acute cholecystitis can present atypically, mimicking acute coronary syndrome (ACS) and leading to diagnostic delays. We report a case of a 49-year-old man with diabetes, hypertension, and dyslipidemia who presented three times over 18 days with episodic chest pain radiating to the neck and back. Serial electrocardiograms and high-sensitivity troponin assays were normal. On the third presentation, he developed epigastric tenderness. A right upper quadrant ultrasound was unremarkable; however, a CT aortogram revealed a 1.7 cm impacted gallstone. A prior normal coronary CT angiogram effectively ruled out significant coronary artery disease. The patient underwent laparoscopic cholecystectomy, confirming acute cholecystitis. This case underscores that biliary pathology should be considered in patients with persistent chest pain and a negative cardiac workup, and cross-sectional imaging is pivotal when ultrasound is inconclusive.

## Introduction

Acute cholecystitis is a common surgical emergency, classically characterized by right upper quadrant (RUQ) pain. A significant diagnostic challenge arises when it presents atypically, mimicking acute coronary syndrome (ACS) [1]. This mimicry is attributable to shared visceral afferent innervation from the gallbladder and heart via the T5-T9 spinal segments, leading to referred pain in the chest [2].

Ultrasonography remains the first-line imaging modality for suspected cholecystitis, but its sensitivity can be compromised by patient-related factors such as obesity or overlying bowel gas [3]. Computed tomography (CT) offers a valuable alternative, with reported sensitivity exceeding 90% for diagnosing acute cholecystitis, particularly when ultrasound findings are inconclusive or discordant with clinical suspicion [4].

We present the case of a middle-aged man with recurrent chest pain initially managed for suspected ACS, where the correct diagnosis of acute cholecystitis was established through cross-sectional imaging after a delayed emergence of abdominal findings.

## Case presentation

A 49-year-old Sri Lankan man with risk factors for coronary artery disease (diabetes mellitus, hypertension, dyslipidemia) presented to the emergency department (ED) with central chest pain radiating to the neck. Of note, a coronary CT angiogram performed eight months earlier for dyspnea on exertion had shown a zero calcium score and no coronary plaque, effectively ruling out significant obstructive coronary artery disease. The pain was described as a heavy sensation, not associated with exertion, diaphoresis, or shortness of breath.

On examination, he was hemodynamically stable. An electrocardiogram (ECG) showed normal sinus rhythm with T-wave inversions in leads III and aVF, consistent with prior tracings and without acute ST-T changes. High-sensitivity troponin was normal (<14 ng/L). His C-reactive protein (CRP) was 2.1 mg/L, and white blood cell count (WBC) was 7.8 x 10^3/uL (Table 1). The chest X-ray was unremarkable. Given his cardiac risk profile, ACS was considered, but with negative serial troponins, he was discharged with follow-up.

**Table 1:** Trend of key laboratory parameters WBC: white blood cell count; Hgb: hemoglobin; CRP: C-reactive protein; ALT: alanine aminotransferase; AST: aspartate aminotransferase; Bilirubin T: total bilirubin; HS Troponin-T: high-sensitivity troponin T.

Labs	ED Visit 1 (Day 0)	ED Visit 2 (Day 15)	ED Visit 3/Admission (Day 18)	Postoperative Day 5	Reference Range
WBC (x10^3/uL)	7.8	8.1	12.4	8.7	4.0 - 10.0
Hgb (gm/dL)	15.2	15.0	17.0	14.6	13.5 - 17.5
CRP (mg/L)	2.1	3.4	93.4	148.6	< 10.0
ALT (U/L)	18	22	30	46	7 - 55
AST (U/L)	15	18	19	29	8 - 48
Bilirubin T (umol/L)	12	14	25	9	< 21
HS Troponin-T (ng/L)	<14	<14	7	-	< 14

Fifteen days later (Day 15), he returned with similar chest pain accompanied by vomiting. His blood pressure was elevated at 161/109 mmHg. ECG showed no dynamic changes. CRP was 3.4 mg/L, and WBC was 8.1 x 10^3/uL (Table 1). Serial troponins remained negative, and he was discharged with a cardiology referral.

Definitive presentation and diagnostic workup

Three days later (Day 18), he presented with worsening symptoms: severe lower chest and upper abdominal pain for two days, associated with vomiting and diaphoresis. The pain radiated to his back. On examination, his blood pressure was markedly elevated at 182/95 mmHg, and his abdomen was soft but now tender in the epigastric region (Table 2). A RUQ ultrasound was unremarkable; no stones were visualized, and the common bile duct was of normal diameter.

**Table 2:** Timeline of patient presentations

Day	Event
Day 0	First ED visit – Chest pain, normal ECG/troponin, discharged
Day 15	Second ED visit – Chest pain with vomiting, normal serial troponins, discharged
Day 18	Third ED visit – Chest pain with epigastric tenderness, admitted

**Figure 1:**
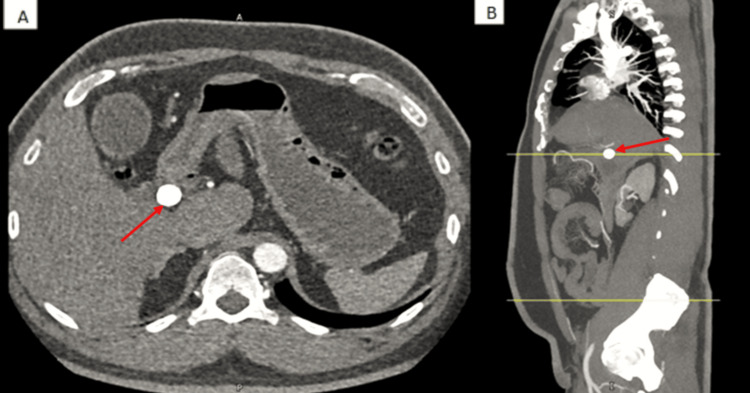
Non-contrast-enhanced CT scan showing a 1.7 cm gallstone impacted at the neck of the gallbladder (red arrows). (A) Axial view and (B) coronal view. There is gallbladder distension without pericholecystic fluid or biliary ductal dilation.

Given the severity of his pain and radiation to the back, a gated CT aortogram was performed to rule out aortic dissection. The stone was visualized on the non-contrast calcium-scoring phase of this study, showing normal aortic opacification with no signs of dissection. However, it incidentally revealed a 1.7 cm gallstone impacted at the gallbladder neck, with gallbladder distension but no clear pericholecystic fluid (Figure 1).

Hospital course

**Figure 2:**
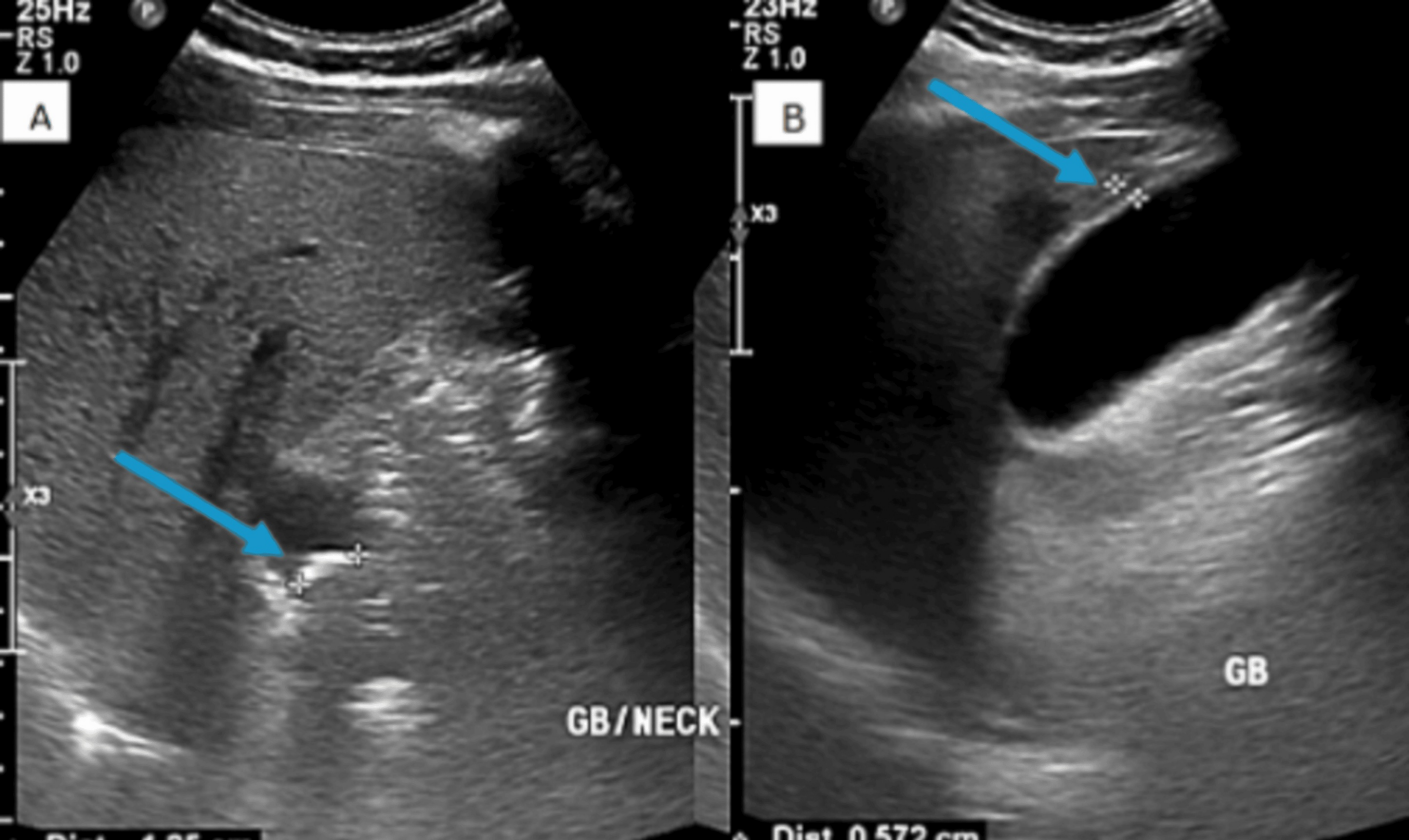
Repeat right upper quadrant ultrasound (longitudinal view). (A) A 1.7 cm obstructing gallstone (arrow) within the gallbladder neck. (B) Thickening of the gallbladder wall measuring 5.7 mm (blue arrow). The liver parenchyma demonstrates diffusely increased echogenicity consistent with hepatic steatosis. No focal hepatic lesions are identified.

The patient was admitted for continued evaluation. On repeat examination, he was now tender in the RUQ region. A focused repeat ultrasound was performed, confirming the presence of a 1.7 cm stone impacted at the gallbladder neck and gallbladder wall thickening measuring 5.7 mm (Figure 2).

**Figure 3:**
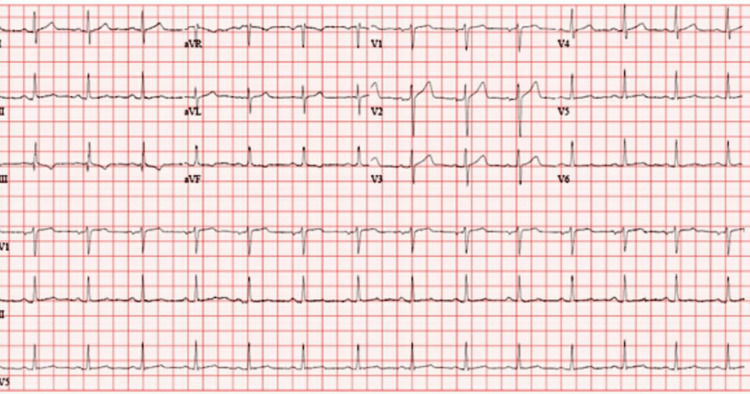
Electrocardiogram (admission day 2) demonstrates normal sinus rhythm with a nonspecific T-wave inversion in lead V6 during an episode of chest pain.

On the second day of admission, he developed severe chest pain, prompting activation of the rapid response team. His ECG remained unchanged (Figure 3), and serial troponins remained negative (Table 1).

The patient was started on intravenous antibiotics (piperacillin/tazobactam 4.5 g every eight hours) and analgesics. Cardiology was consulted, and given his prior normal coronary CT angiogram (zero calcium score, no plaque), ACS was deemed unlikely. His symptoms were attributed to confirmed gallbladder pathology.

A laparoscopic cholecystectomy was performed, revealing a thick-walled, inflamed gallbladder with an impacted stone. His postoperative recovery was uneventful, and he was discharged on the fourth postoperative day. CRP on postoperative day 5 was 148.6 mg/L (Table 1), which was an expected acute phase response to surgery, as the patient was afebrile and clinically improving.

## Discussion

This case of a patient with recurrent chest pain, initially attributed to suspected ACS, diagnosed with acute cholecystitis, highlights a critical diagnostic challenge: biliary pathology can mimic cardiac ischemia, leading to delayed diagnosis and intervention even in patients with cardiac risk factors. Several key insights emerge from this case.

First, the absence of RUQ tenderness does not exclude acute cholecystitis. The patient's initial chest and back pain, without abdominal tenderness, aligns with the phenomenon of referred pain from the gallbladder. This is mediated by shared visceral afferent fibers from the gallbladder and the heart that converge at spinal cord segments T5-T9 [2]. Pain from the inflamed gallbladder can be misinterpreted by the central nervous system as originating from the chest. The later development of epigastric and RUQ tenderness, occurring over several days, represents the progression from visceral to somatic pain as the inflammation extended to involve the adjacent parietal peritoneum [1].

Second, a negative initial ultrasound has poor negative predictive value in atypical presentations. While ultrasonography remains the first-line imaging modality for suspected cholecystitis, its sensitivity can be compromised by patient-related factors such as obesity, overlying bowel gas, or an impacted stone [3]. In this case, the initial RUQ ultrasound was falsely negative. In patients with persistent symptoms and a negative cardiac workup, cross-sectional imaging (CT) should be considered earlier. CT has a reported sensitivity exceeding 90% for diagnosing acute cholecystitis and is particularly valuable when ultrasound findings are inconclusive or discordant with clinical suspicion [4]. The rising CRP from 2.1 to 93.4 mg/L, despite normal troponins, served as a clinical trigger to pursue advanced imaging, leading to the diagnosis.

Third, normal cardiac biomarkers do not rule out biliary pathology. Unlike prior reports where cholecystitis was associated with troponin elevation due to systemic inflammation or coronary vasospasm [5,6], our patient's troponin remained normal. This, combined with a prior normal coronary CT angiogram (zero calcium score, no plaque), was instrumental in confidently steering the clinical team away from ACS and toward alternative diagnoses. Acute cholecystitis has been documented to masquerade as myocardial infarction, with case reports describing patients who underwent extensive cardiac evaluation before the correct diagnosis was established [7].

Finally, the postoperative CRP elevation (148.6 mg/L) was an expected acute phase response to surgery, not a sign of worsening infection, as the patient was afebrile, hemodynamically stable, and clinically improving. This distinction is important to avoid unnecessary antibiotic escalation.

This case exemplifies the diagnostic complexity of acute cholecystitis when it presents with cardiac-like symptoms. It reinforces the necessity for clinicians to consider biliary pathology in patients with persistent, unexplained chest pain, even in the absence of classic abdominal signs or positive cardiac biomarkers. A high index of suspicion, combined with the judicious use of cross-sectional imaging and repeated clinical reassessment, is paramount to avoid diagnostic delays and ensure optimal patient outcomes.

Learning points

(i) A broad differential should be maintained in patients with chest pain and a negative cardiac workup, especially when pain radiates to the back or is atypical, consider non-cardiac etiologies such as biliary pathology. (ii) Referred pain patterns should be recognised. The gallbladder and heart share visceral innervation (T5-T9). Gallbladder disease can be present solely with chest, back, or shoulder pain. The absence of RUQ tenderness does not rule out cholecystitis, especially early in its course. (iii) Imaging should be utilized strategically. When clinical suspicion for cholecystitis remains high, but the initial ultrasound is inconclusive, cross-sectional imaging (CT) is a valuable problem-solving tool that can both identify the diagnosis and exclude life-threatening differentials. A rising CRP with negative cardiac markers may trigger earlier advanced imaging. (iv) Re-evaluation should be done dynamically. As a patient's symptoms evolve, so should the differential diagnosis. Persistent or worsening symptoms warrant a reconsideration of the initial clinical impression and diagnostic strategy.

## Conclusions

This case demonstrates that acute cholecystitis can be a great mimicker, presenting solely with recurrent chest pain and delaying diagnosis. It highlights the critical importance of maintaining a broad differential diagnosis, recognizing patterns of referred visceral pain, and strategically employing cross-sectional imaging to solve complex diagnostic puzzles. A high index of suspicion for biliary disease in patients with persistent, unexplained chest pain is essential to avoid diagnostic delays and associated morbidity.
